# Endomyocardial biopsies in patients with left ventricular hypertrophy and a common Chinese later-onset fabry mutation (IVS4 + 919G > A)

**DOI:** 10.1186/1750-1172-9-96

**Published:** 2014-07-01

**Authors:** Ting-Rong Hsu, Shih-Hsien Sung, Fu-Pang Chang, Chia-Feng Yang, Hao-Chuan Liu, Hsiang-Yu Lin, Chun-Kai Huang, He-Jin Gao, Yu-Hsiu Huang, Hsuan-Chieh Liao, Pi-Chang Lee, An-Hang Yang, Chuan-Chi Chiang, Ching-Yuang Lin, Wen-Chung Yu, Dau-Ming Niu

**Affiliations:** 1 Institute of Clinical Medicine, National Yang-Ming University, Taipei, Taiwan; 2 Department of Pediatrics, Taipei Veterans General Hospital, No. 201, Section 2, Shih-Pai Road, 112 Taipei, Taiwan; 3 Division of Cardiology, Department of Medicine, Taipei Veterans General Hospital and National Yang-Ming University, School of Medicine, No. 201, Section 2, Shih-Pai Road, 112 Taipei, Taiwan; 4 Pathology and Laboratory Medicine Department, Taipei Veterans General Hospital, Taipei, Taiwan; 5 Department of Pediatrics, Mackay Memorial Hospital and Department of Medicine, Mackay Medical College, Taipei, Taiwan; 6 Taiwan Clinical Trial Consortium in Fabry Disease, Taipei, Taiwan; 7 Neonatal Screening Center, Chinese Foundation of Health, Taipei, Taiwan; 8 College of Medicine, China Medical University, Taichung, Taiwan

**Keywords:** Endomyocardial biopsy, Enzyme replacement therapy, Fabry disease, IVS4 + 919G > A, Left ventricular hypertrophy

## Abstract

**Background:**

In Taiwan, DNA-based newborn screening showed a surprisingly high incidence of a cardiac Fabry mutation (IVS4 + 919G > A). The prevalence of this mutation is too high to be believed that it is a real pathogenic mutation. The purpose of this study is to identify the cardiac pathologic characteristics in patients with left ventricular hypertrophy and this mutation

**Methods and results:**

Endomyocardial biopsies were obtained in 22 patients (Median age: 61, males: 17; females: 5) with left ventricular hypertrophy and the IVS4 + 919G > A mutation; five patients had not received enzyme replacement therapy (ERT) before biopsy, while the other 17 patients had received ERT from 8 months to 51 months. Except for three patients who had received ERT for more than 3 years, all other patients showed significant pathological change and globotriaosylceramide (Gb3) accumulation in their cardiomyocytes. In contrast to classical Fabry patients, no Gb3 accumulation was found in the capillary endothelial cells of any of our patients. Fourteen patients (63.6%) were found to have myofibrillolysis.

**Conclusions:**

All of the untreated and most of the treated IVS4 + 919G > A patients showed typical pathological changes of Fabry disease in their cardiomyocytes. No endothelial accumulation of Gb3 was found, which is similar to the findings of several previous reports regarding later-onset Fabry disease. This result highly suggests that the IVS4 + 919G > A is a real pathogenic later-onset Fabry mutation.

## Background

Fabry disease (MIM 301500) is an X-linked lysosomal storage disorder resulting from deficient alpha-galactosidase A (α-Gal A) activity. The estimated incidence of classic Fabry disease is 1 in 40,000-60,000 males in the general population [1]. The deficient α-Gal A activity results in progressive accumulation of glycosphingolipid, predominantly globotriaosylceramide (Gb3), in the walls of small blood vessels, nerves, dorsal root ganglia, renal glomerular and tubular epithelial cells, and cardiomyocytes. Clinical features in classically affected patients include acroparesthesia, angiokeratoma, and hypohidrosis in early childhood or adolescence and progress to renal insufficiency, cardiomyopathy, and cerebrovascular disease in adulthood [2-6]. Recently, several different later-onset phenotypes of Fabry disease have been identified, which have drawn the attention of more physicians [7-9]. Patients with later-onset Fabry disease have higher residual enzyme activities than those of the classical type. They lack the classic symptoms of Fabry disease and present relatively fewer or isolated symptoms such as hypertrophic cardiomyopathy, renal failure, or cryptogenic stroke at later stages in life [10-14]. The cardiac later-onset phenotype usually presents only with cardiac manifestations, such as hypertrophic cardiomyopathy, mitral insufficiency and/or arrhythmias in the fifth to eighth decade [12,15,16].

**Figure 1:**
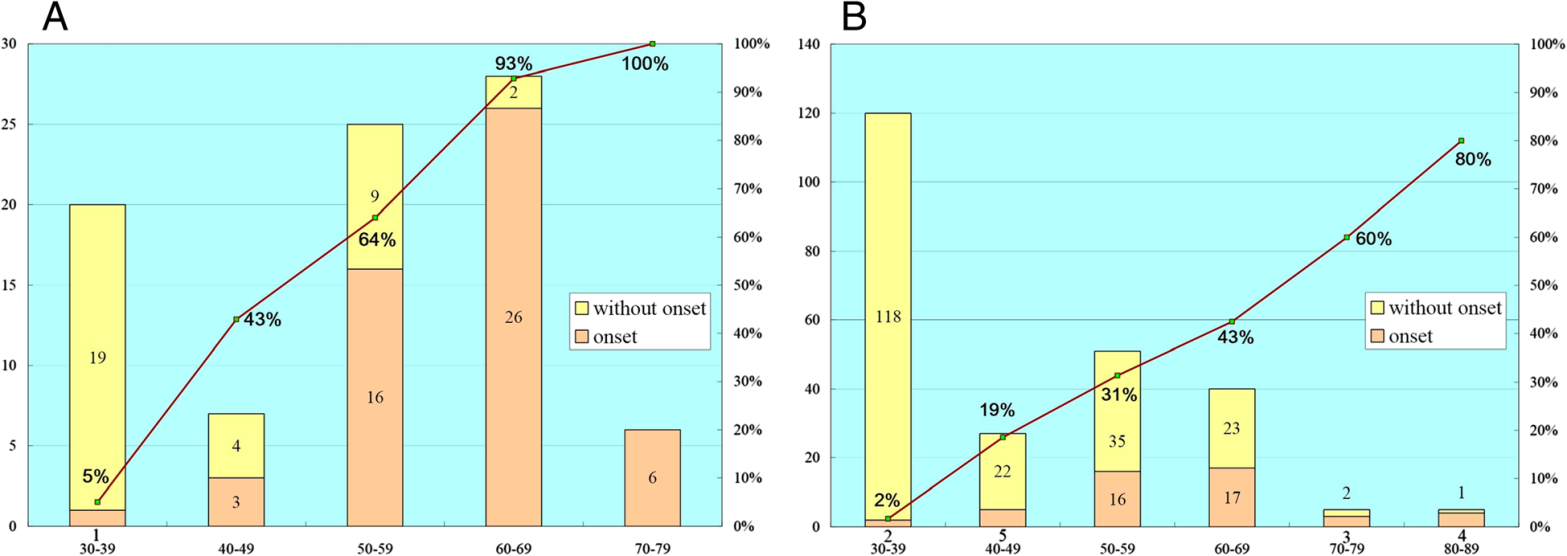
**Disease onset rate in male and female adults with IVS4 + 919G > A.** The figure showed the disease onset rate according to the ages of male and female adults (>30y/o) with IVS4 + 919G > A mutation in males 1**(A)** and females 1**(B)**. The X-axis showed the age distribution and the Y-axis showed the patient numbers. The disease onset rate increased gradually, as patients got older for both males and females.

In Taiwan, our team first revealed a surprisingly high incidence (approximately 1 in 1600 males) of a later-onset GLA mutation, IVS4 + 919G > A (GenBank accession nos. CS020811), in our population [17] and also identified this mutation in a number of Taiwan Chinese adult patients with idiopathic hypertrophic cardiomyopathy [18]. Thereafter, another newborn screening center of Taiwan also revealed a very similar incidence (1 in 1,460 males) of this mutation in their study [19]. More recently, DNA-based newborn screening for this mutation revealed a higher incidence (1/875 in males and 1/399 in females) than our previous enzyme-based Fabry newborn screening in Taiwan [20]. Because of the high prevalence of this IVS4 + 919G > A mutation in our population [21,22], we analyzed the disease onset rate according to the ages of the male and female adults with this mutation. 86 male and 248 female adults who were older than 30 years were enrolled in this analysis. We found the disease onset rate for both male and female patients increased gradually with increasing age (Figure 1). Around 77% of male adults and 35% of female adults, who were older than 40 years old, had already developed hypertrophic cardiomyopathy. In particular, 94%(32/34) of the males who were older than 60 years old and 100% (6/6) who were older than 70 years old showed significant hypertrophic cardiomyopathy (Figure 1). We believe this result strongly suggests that this IVS4 + 919G > A is a real pathogenic mutation. Although, the IVS4 + 919G > A mutation was found in such a high frequency, the pathologic changes in later-onset Fabry disease with this specific mutation is still poorly known. It is important to look into the pathologic characteristics and changes of cardiomyopathy in these patients [23].

Due to the high prevalence of IVS4 + 919G > A mutation in our population, a new treatment guideline has been set up by the Bureau of National Health Insurance in Taiwan. All Fabry patients applying for enzyme replacement therapy (ERT) funding from the national health insurance have to receive an endomyocardial biopsy examination to demonstrate that Fabry disease is the primary cause of their hypertrophic cardiomyopathy. This guideline gives us the opportunity to observe a large series of cardiac pathologic changes in these patients with left ventricular hypertrophy and the IVS4 + 919G > A mutation.

## Patients and methods

### Patient population

A total of 22 Fabry patients (17 males and 5 females, median age: 61 years old) with left ventricular hypertrophy carrying the IVS4 + 919G > A mutation were enrolled in this study. The demographic data, laboratory results, image results and pathologic findings of the endomyocardial biopsies were collected retrospectively from December 2012 to April 2013.

Five patients (nos.1-5) did not receive ERT before endomyocardial biopsy. The others had received agalsidase alfa (Replagal®, Shire) 0.2 mg/kg every other week as the enzyme replacement therapy for at least 8 months (range 8-40 months). Two patients (nos. 16 and 17) had initially received agalsidase beta (Fabrazyme®, Genzyme) 1 mg/kg every other week and then changed to Replagal® 0.2 mg/kg in 2009 owing to the global shortage of Fabrazyme.

The study complies with the Declaration of Helsinki and is approved by the medical ethics committee of Taipei Veteran General Hospital. Written informed consents were obtained.

### GLA gene mutation

Blood samples were obtained from these patients in blood collecting tubes containing ethylene diamine tetraacetic acid, and samples were stored at 4°C. DNA was isolated from whole blood using the GFX genomic Blood DNA Purification Kit (Amersham Biosciences, UK) following the manufacturer’s instructions. The primer sets were used to amplify the sequences of seven GLA exons and the region including IVS4 + 919G > A [24,25]. The polymerase chain reaction products were analyzed by 1.5% agarose I (Amresco) gel electrophoresis and then eluted in the polymerase chain reaction Advanced PCR Clean Up System (Viogene, USA.). Direct sequencing of the α-Gal A gene was processed using the BigDye Terminator v3.1 Cycle Sequencing Kit (Applied Biosystems) and ABI Prism 3730 Sequencer [26].

### Plasma α-Gal A activity and Plasma LysoGb3

Plasma α-Gal A activity was determined using the substrate 4-methylumbelliferyl α-D-galactopyranoside (5 mmol/L) freshly prepared in 117 mmol/L N-acetyl-D-galactosamine/50 mmol/L citric-phosphate buffer, pH 4.6, before every assay. In brief, 50 μL of plasma was mixed with 300 μL of the substrate solution, incubated at 37°C for 2 hours, and 0.2 N glycine-NaOH was added to stop the reaction. Fluorescence intensity was measured with the excitation and emission wavelengths of 365 and 450 μm, respectively [27,28]. Plasma LysoGb3 was detected by tandem mass spectrometry that was performed in positive ion mode (ES+) on a triple quadruple mass spectrometer (Quattro Ultima, Waters, Milford, MA) with NeoLynx software version 4.1. A multiple reaction monitoring (MRM) mode was used for the measurement of lysoGb3. The analyzing methods were modified from the protocol provided by Shire [29].

### Echocardiography

Echocardiography was performed routinely for the Fabry patients to check the hypertrophic cardiomyopathy. Left ventricular mass was calculated according to the Devereux cube formula and indexed to height^2.7^ (LVMI). Left ventricular hypertrophy by echocardiography was defined by a LVMI of >47 g/m^2.7^ in women or >50 g/m^2.7^ in men [30-32]. To avoid variability of LMVI measurements, only the results performed by two cardiologists specializing in echocardiography and measured with the same machine and protocol were used in this study.

### Cardiac magnetic resonance imaging

Magnetic resonance imaging (MRI) of the heart was carried out as part of stand assessment on a 1.5 T scanner (Excite II; GE medical systems, Milwaukee, WI). Late enhancement (LE) technique by gadolinium-contrast cardiac MRI examination was applied for the later-onset Fabry patients for assessment of fibrosis. The participants underwent a scan with spin echo double IR T1WI and T2WI pulse sequences and Fiesta pulse sequence, on axial, coronal, and variable scanning plans. Images were acquired 10 minutes after the intravenous injection of gadolinium. The myocardial delayed enhancement protocol included the pre-Gd axial FIESTA cine and grid tagging in 8 mm slice, and the post-Gd myocardial delayed enhancement of short axis/four-chamber views in 8-mm slice and oblique axial cine of ascending aorta in 8-mm slice to detect changes in tissue integrity in the left ventricle myocardium [33-35]. Severe myocardial fibrosis was defined by at least two affected left ventricular segments, which are followed by the American heart association guideline [36], and mild myocardial fibrosis was defined by one segment affected.

### Cardiac catheterization and endomyocardial biopsy

Right heart catheterization was approached via the right internal jugular vein under digital X-ray guidance. A flexible endomyocardial bioptome was inserted into the right ventricle and 2-3 specimens were obtained from interventricular septum and submitted to histological examination.

### Histological studies

Cardiac specimens selected for light microscope were fixed in 10% buffered formalin, embedded in paraffin. The sections were cut from the paraffin block and stained with haematoxylin and eosin (H&E), and Masson’s trichrome. Cardiac specimens selected for electron microscope were fixed in 2.5% glutaraldehyde in phosphate buffer, post-fixed with 1% OsO4 in Sorenson’s phosphate buffer, followed by dehydration through a graded series of ethanol washes, and embedded in Spurr’s EPON. Semithin sections were cut from the block and stained with toluidine blue for adequate preview under a microscope. Ultrathin sections were prepared and examined under an electron microscope.

In the idealized model of a cardiomyocyte, as an elliptic cylinder, different diameters such as major axis (longest diameter) or minor axis (shortest diameter), etc. are measured, even in one transverse section of a cardiomyocyte. The current study revealed that, in the transverse section, the ratio between the major axis and minor axis of the cardiomyocytes is usually around 1.1 (11/10) [37]. Therefore, the diameters of cardiomyocytes in transverse section can be approximately estimated. Because the minor axis of the transverse section of cardiomyocytes can be measured more easily and reliably than other diameters of the other axis, the diameter of the minor axis of the transverse section of cardiomyocytes was used to present the diameter of cardiomyocytes in several previous reports [38-41]. In accordance with these previous reports, we measured the minor axis at the nuclear level in transverse section to present the diameter of cardiomyocytes of our patients in this study. A total of 15 cells from 3 micrographs were measured for each sample. Control samples were retrospectively obtained from 8 anonymous patients who were provided by the department of pathology in our medical center. All the samples were obtained during the electrophysiology study for arrhythmia. None of these patients had any evidence of hypertrophic cardiomyopathy. All the histopathologic specimens were blindly reviewed by the same pathologist.

### Data analysis and statistics

The data in some parts of the study were analyzed with descriptive statistics (mean, standard deviation). Due to the small number of patients and the uncontrolled nature of this study, no inferential statistics were used. Results are presented as actual measurements from individual patients. SPSS Version 20 (SPSS Inc, Chicago, Illinois, USA) was used for descriptive statistics.

## Results

The demographic data and clinical manifestations of these patients are shown in Table 1. Interestingly, high prevalences of hypertension and hyperlipidemia were noted in these IVS4 + 919G > A patients with hypertrophic cardiomyopathy. Out of these 22 patients, 12 (54.5%) were noted to have hypertension. All of these hypertensive patients received anti-hypertensive treatment and were kept normotensive (<140/90 mmHg) for at least 6 months before commencement of ERT. Seven (31.8%) patients were found to have hyperlipidemia and all of them were well controlled by medication for at least 6 months.

**Table 1:** The demographic data and clinical manifestations of the Fabry disease patients with IVS4 + 919G > A mutation

**Pt**	**Age**	**Sex**	**Comorbidity**	**Histology**^**a**^					**α-Gal A**^**c**^	**LysoGb3**^**d**^	**LVMI**^**e**^		**Cr/eGEF**^**f**^	**LE**^**g**^	**ERT**^**i**^**/Dose**^**j**^
				**HC**	**MB**	**MFL**	**Other**	**Diameter**			**Initial**	**biopsy**			
1	58	M	Hyperlipidemia; Hypertension	+	+	+		34.7 ± 4.4	1.35	3.21	57.0	57.0	0.88/94.5	No	Not yet/0
2	61	M	Hyperlipidemia; Hepatitis B	+	+	+		21.8 ± 3.3	2.75	6.51	66.9	66.9	0.92/88.9	severe	Not yet/0
3	75	M	Coronary artery disease; Arrhythmia; Hepatitis C; Cholecystitis	+	+	+		31.5 ± 4.0	1.15	6.51	83.0	83.0	1.29/57.7	NA^h^	Not yet/0
4	66	M	DM; Hypertension;ESRD s/p renal transplantation; Cholecystitis; Hyperuricemia	+	+	+		30.8 ± 5.6	1.29	7.13	154.9	154.9	1.77/41.1	severe	Not yet/0
5	64	M	Hypertension; Coronary artery disease; Hyperlipidemia; Hyperuricemia	+	+	+		33.5 ± 8.0	1.32	7.47	128.1	128.1	0.97/82.8	No	Not yet/0
6	58	M	Hyperlipidemia	+	+	-		24.1 ± 2.4	2.17	9.60	54.5	38.5	1.01/80.6	No	8M/3.80
7	61	M	Cubital tunnel syndrome	+	+	+		25.9 ± 2.8	1.54	7.53	52.2	52.2	0.85/97.4	severe	10M/4.11
8	56	M	Hypertension; DM; Duodenal ulcer	+	+	-		25.6 ± 7.0	1.39	6.13	74.5	74.8	0.92/90.5	No	10M/4.35
9	66	M	Hyperlipidemia; Sick sinus syndrome	+	+	+	Fatty^b^	24.9 ± 3.6	1.15	9.77	136.4	113.9	1.4/53.9	No	1Y5M/7.53
10	52	M	Coronary artery disease; Fatty liver	+	+	+	Fibrosis	21.1 ± 5.0	0.94	11.27	54.8	61.0	0.86/99.3	No	1Y6M/8.18
11	62	M	BPH; Bladder cancer; Osteoarthritis	+	+	-		21.6 ± 4.0	0.79	5.06	50.7	52.8	0.95/85.4	mild	1Y11M/9.76
12	63	M	Hyperlipidemia; Arrhythmia s/p pacemaker	+	+	-		23.2 ± 3.4	1.02	13.51	51.2	50.9	1.16/67.6	NA^h^	2Y9M/14.90
13	58	M	Hypertension; Hyperlipidemia	+	+	+		26.2 ± 5.6	1.32	5.44	50.5	41.0	1.15/69.4	mild	2Y10M/15.61
14	58	M	Hypertension	+	+	+		24.0 ± 5.4	1.30	8.22	66.5	83.7	1.1470.1	No	2Y11M/16.33
15	66	M	Hypertension, Hyperuricemia	+	+	+		24.2 ± 4.1	1.47	14.54	88.2	81.4	0.81/101.3	severe	2Y11M/16.33
16	47	M	Hypertension; Arrhythmia	+	Scanty	-		23.1 ± 4.1	0.65	6.16	92.1	74.9	0.8/110.1	severe	4Y3M/44.84
17	58	M	Hypertension; DM; BPH	+	No	-		23.6 ± 3.8	0.88	5.19	76.0	71.4	0.84/99.8	severe	4Y3M/36.13
18	60	F	Lung adenocarcinoma; Hypertension; Insomnia	+	+	-		24.6 ± 3.4	5.79	2.12	48.9	31.2	0.75/83.8	No	1Y2M/6.51
19	65	F	Hypertension	+	+	+		20.5 ± 3.3	9.63	4.24	52.1	37.2	0.62/102.7	No	2Y2M/12.34
20	70	F	Hypertension; Major depression	+	+	+		25.8 ± 6.9	6.51	3.67	64.0	54.1	0.67/92.5	mild	2Y9M/14.90
21	62	F	Hypertension	+	+	+	Fatty^b^	23.5 ± 3.5	5.17	3.49	53.4	31.1	0.83/74.0	mild	2Y10M/15.35
22	61	F	Chronic renal insufficiency; Hyperuricemia	+	Scanty	-		23.5 ± 7.1	4.03	2.60	59.2	49.0	2.98/17.0	No	3Y1M/18.50

DM: diabetes mellitus, BPH: benign prostate hyperplasia, ERT: enzyme replacement therapy, ESRD: end-stage renal disease, MRI: magnetic resonance imaging. ^a^Histology of endomyocardial biopsies: HC: hypertrophic cardiomyocyte; MB: myelin bodies; MFL: myofibrillolysis; Diameter: minor cardiomyocyte diameter in mean ± SD (μm), and the control: 14.89 ± 2.63 μm; ^b^Fatty: fatty infiltration within the myocardium; ^c^α-Gal A: normal reference ranges: 7.9-16.9 nmol/hr/ml; ^d^LysoGb3: normal reference ranges:<0.5 nM; ^e^LVMI (g/m^2.7^): left ventricular mass index; ^f^Cr/eGFR: Cr(serum creatinine): normal range: 0.5-1.5 mg/dl; eGFR (estimated glomerular filtration rate): normal range: >60 ml/min/1.73 m^2^; ^g^LE: late-enhancement by MRI: No: no fibrosis, mild: one segment, severe: more than 2 segment; ^h^NA: not available. The two patients had arrhythmia with implanted pacemaker and MRI cannot be arranged; ^i^ERT duration before biopsy; ^j^Dose (mg/kg): the cumulative dose of ERT before endomyocardial biopsy.

**Figure 2:**
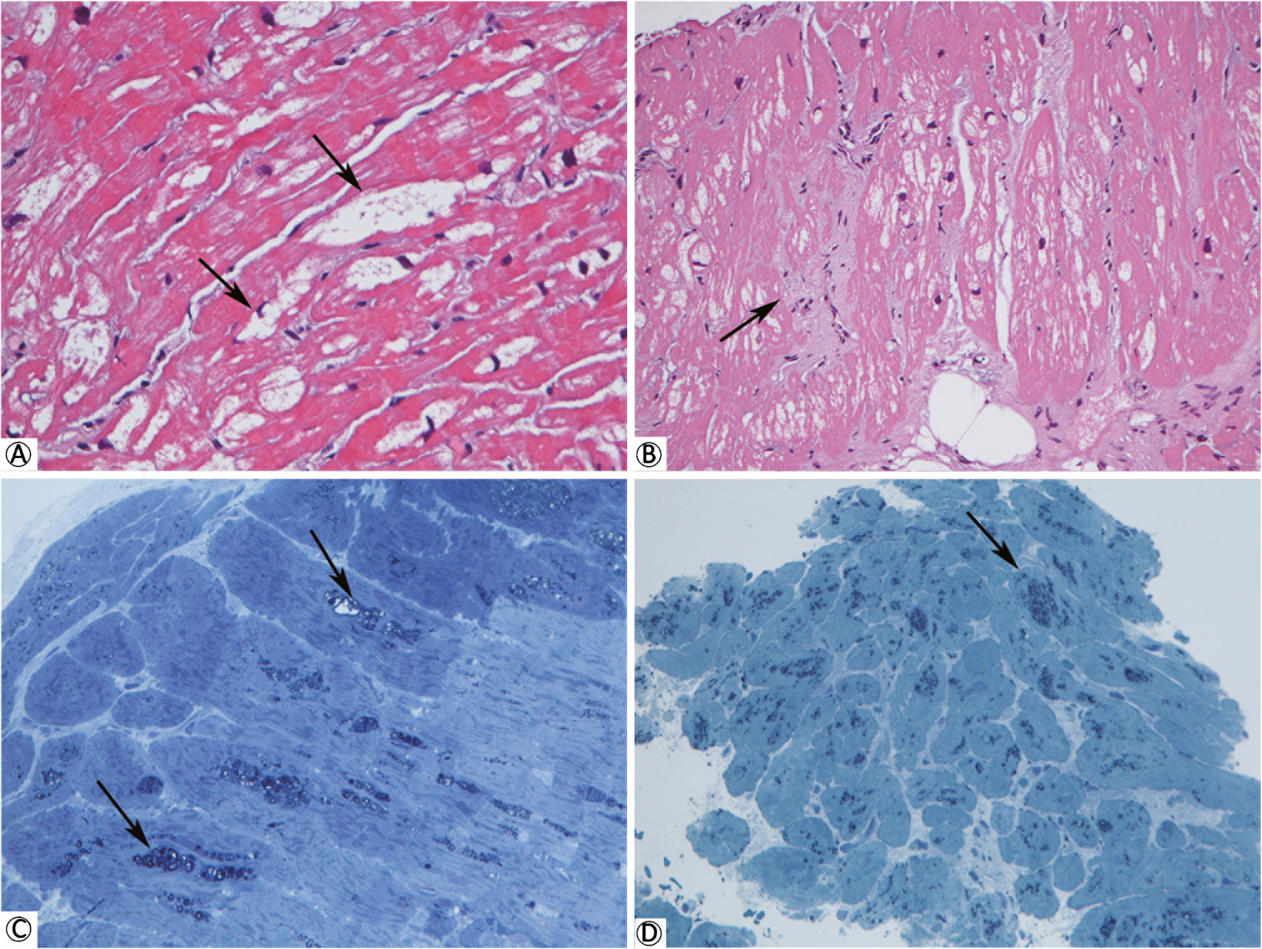
**Histologic examination of the cardiomyopathic patients with IVS4 + 919G > A mutation. (A)** Endomyocardial biopsy of patient no. 4, who had not received ERT, showed a diffuse vacuolization (arrows) attributed to the lysosomal Gb3 storage and hypertrophic change of cardiomyocytes with hematoxylin and eosin (H&E) staining. These findings were found in most of the enrolled patients. **(B)** H&E staining of the endomyocardium showed mild interstitial fibrosis (arrow) only in one patient (no. 10) and showed similar diffuse vacuolization. Toluidine blue staining of the endomyocardium revealed cytoplasmic granular inclusion (arrow), which is consistent with Gb3 accumulation, in patient no. 4 **(C)** and patient no. 12 **(D)** who has received ERT for 2 year and 9 months.

**Figure 3:**
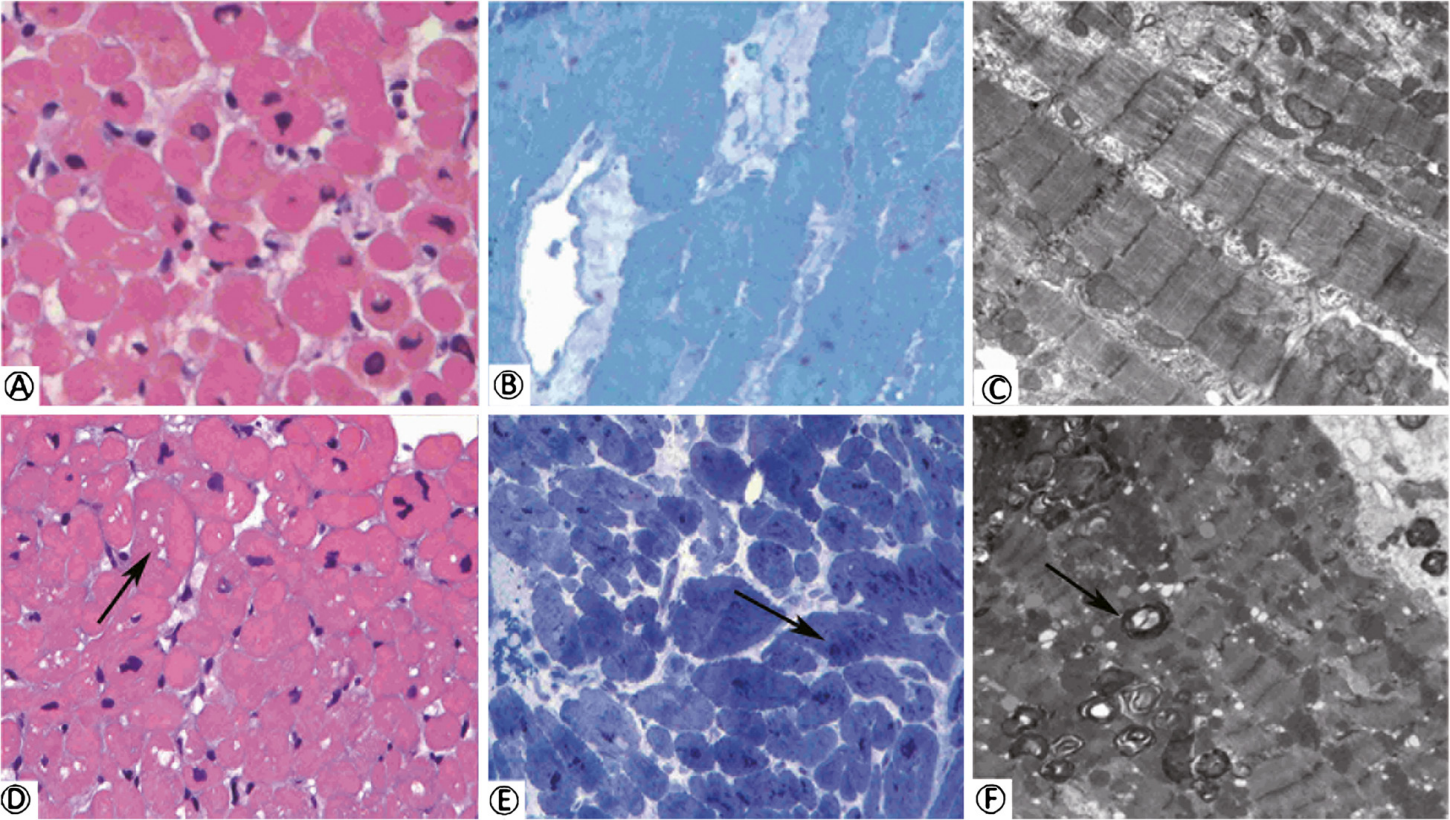
**Scanty or little Gb3 accumulation of the two patients who had received ERT.** The figures showed the endomyocardial histologic examination of the patients (No. 16 and 17) who had received ERT for more than 3 years. Patient No. 17 showed no Gb3 accumulation with H&E staining **(A)**, toluidine blue staining **(B)**, and electron microscope exam **(C)**. Patient No. 16 still showed scanty vacuolization (arrow) with H&E staining **(D)**, Gb3 accumulation (arrow) with Toluidine blue staining **(E)**, and myelin bodies (arrow) in electron microscope exam (**F)**.

**Figure 4:**
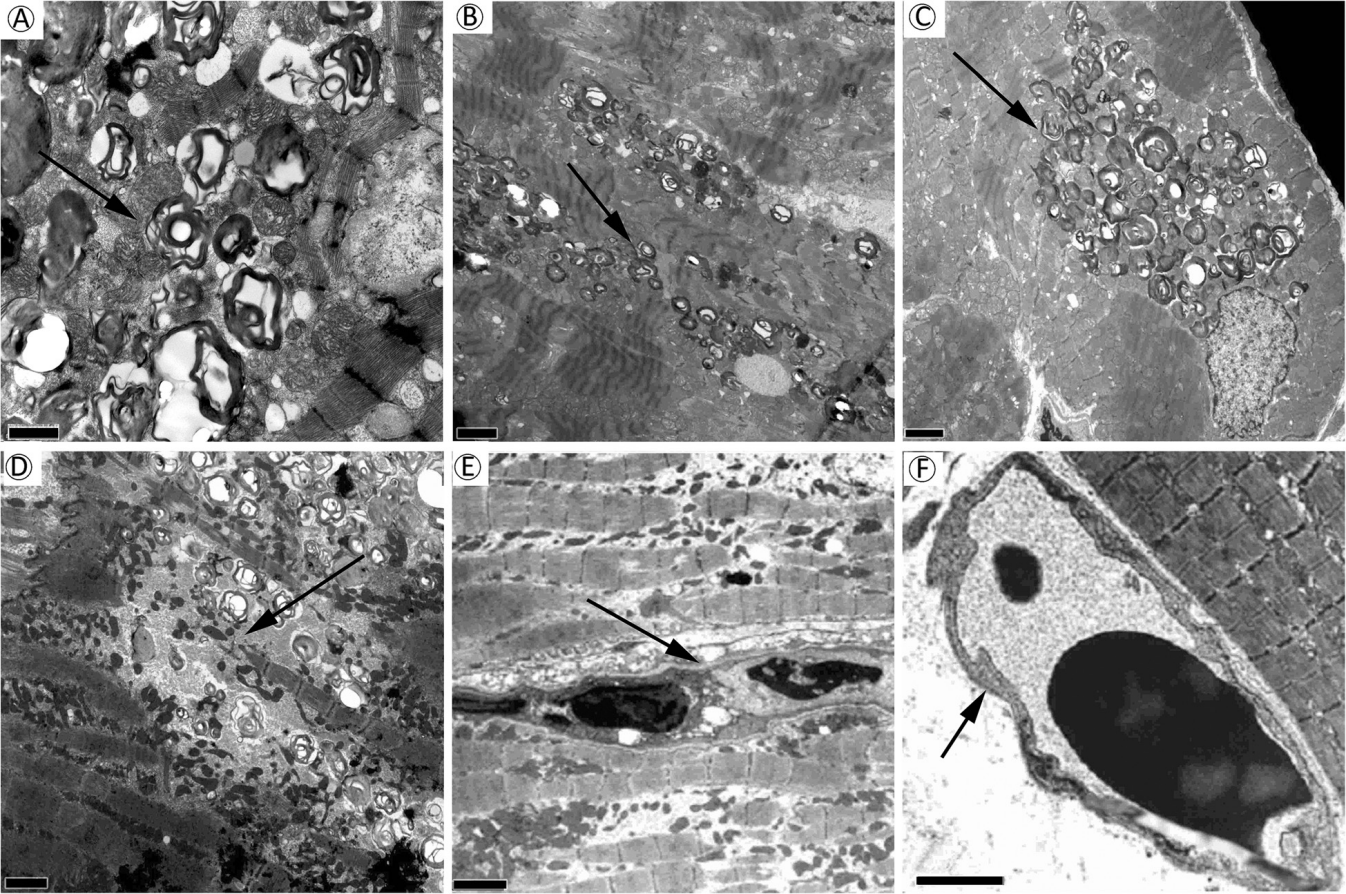
**Electron microscopic examination of the patients with left ventricular hypertrophy and IVS4 + 919G > A mutation.** Electron microscopic findings of the cardiomyocytes from patient no. 20 showed abundant membrane-bound lamellar myelin bodies (“zebra” or “onion-skin” appearance) (arrow) in this figure **(A)**. The myelin bodies (arrow) were also found in most of the patients who received biopsy in this study. Further examples were showed in figure **(B)** (patient no. 8) and **(C)** (patient no. 2). Focal loss of myofilament (myofibrillolysis) (arrow), which may be attributed from glycosphinogolipid accumulation and was found in 14 out of 22 case, is presented in figure **(D)** (patient no. 4). No Gb3 accumulation was found in the capillary endothelium of all these patients. The endothelium (the arrow) were presented in figure **(E)** (patient No. 2) and **(F)** (patient No. 3). The scale bars = 2 μm.

Twenty out of 22 patients received gadolinium-contrast enhancement cardiac MRI with LE technique and 6 patients (30.0%) were found to have severe fibrosis which is defined as at least 2 segments that are positive in myocardium delayed enhancement scan.All the endomyocardial biopsies were successfully performed without complication. For the pathologic change of the endomyocardial biopsies, all 22 patients (100%) showed myocardial hypertrophic change with distorted nuclei, and increased cardiomyocyte size with H&E staining. Five (100%) of the patients who did not receive ERT showed diffuse vacuolization in their myocardiocytes (Figure 2A) which is a common pathologic finding for Gb3 accumulation in cardiac biopsies of Fabry patients. In the other 17 patients who had received ERT, except for 3 patients (nos. 16, 17 and 22) who had received ERT for more than 3 years (Figure 3), all of them also showed diffuse Gb3 accumulation in cardiomyocytes. Regarding the toluidine blue staining, except those 3 patients (nos. 16, 17 and 22), the other 19 patients were also noted to have a lot of dark blue substance accumulation in cardiomyocytes, which is consistent with Gb3 accumulation (Figure 2C and D). In addition to Gb3 accumulation, patient no. 10 was found to have interstitial fibrosis among the cardiomyocytes with H&E staining (Figure 2B). Regarding the electron microscopic examination, except for those three patients, all other patients were found to have abundant lamellar myelin bodies in their cardiomyocytes (Figure 4). Furthermore, 14 patients (63.6%) were found to have significant myofibrillolysis (Figure 4D). There was no Gb3 accumulation in the capillary endothelium of any of these patients (Figure 4E, and F), which is compatible with the histopathologic finding of later-onset Fabry disease.

**Figure 5:**
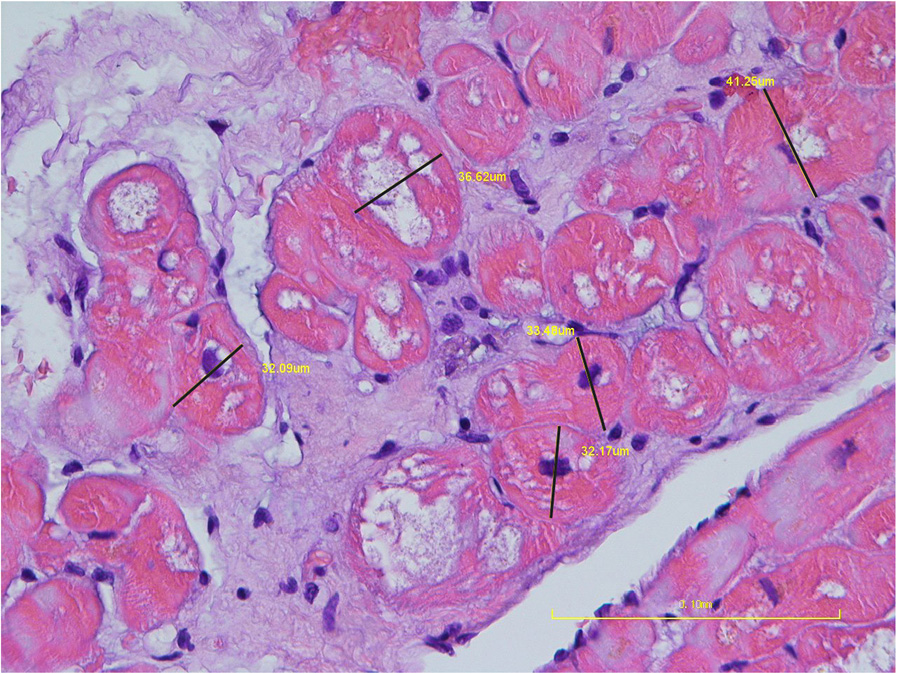
**The cardiomyocyte diameter of the patient no. 1 with H&E staining.** Diameters (minor axis) of the cardiomyocyte in transverse sections were obtained by measuring the shortest perpendicular measurement line (dark line), which was placed at the level of nucleus. Five cardiomyocytes were measured and their diameters were also presented in beside the dark line (yellow).

The diameters of cardiomyocytes were measured in all 22 patients (Figure 5). The average diameter of the cardiomyocytes was 25.35 ± 6.02 μm. The result of each individual is shown in Table 1. The average diameter of the control cardiomyocytes was 14.89 ± 2.63 μm.

## Discussion

Recently, the later-onset phenotypes of Fabry disease were found to be about 10 times more frequent in most populations [1,20,42,43]. Owing to the characteristics, the terminology “later-onset” Fabry disease might be more appropriate than “variant” Fabry disease. In fact, an increasing number of novel mutations have been found and believed to be later-onset mutations through high-risk patient screenings or newborn screenings. On the other hand, some mutations, which were previously thought to be later-onset mutations, have been proved to be non-pathogenic. The most noticeable example is the p,E66Q mutation (c.196G > C), a previously identified renal later onset type mutation, which has a very high prevalence rate in Japanese and Korean populations (0.5-1%) and which has now been proved by pathologic studies to be merely a single nucleotide polymorphism, not a pathogenic mutation [44]. Therefore, it is insufficient to confirm a later onset mutation based on its enzyme activity and clinical manifestations such as hypertrophic cardiomyopathy or renal impairment.

Biomarkers, such as Gb3 or lyso-Gb3 were considered to be helpful to determine if the novel mutation is a true pathogenic mutation [27]. However, several reports indicated that Gb3, and even lysoGb3 might be normal in certain Fabry patients who had developed significant clinical manifestations with the pathogenic mutations [45,46]. Renal or cardiac biopsy can provide more information to identify if a novel mutation is a real pathogenic mutation. In the retrospective single-center direct observational study, we reviewed the endomyocardial biopsies of large numbers of later-onset Fabry patients with IVS4 + 919G > A mutation to prove this mutation is a real pathogenic mutation.

Cardiac biopsy of classical Fabry disease patients revealed significant Gb3 accumulation in both endothelium and cardiomyocytes [47-53]. Furthermore, the degree of endothelial Gb3 deposition was even used as a scoring system for monitoring the long-term cardiac therapeutic outcomes of ERT in classic Fabry disease (Thurberg, B.L. et al) [54]. In contrast to classical Fabry disease, cardiac biopsy of the later-onset patients revealed that Gb3 accumulation was confined to the myocardium and did not involve endothelial cells [55]. Other reports have also showed no endothelial Gb3 accumulation in later-onset Fabry disease [56-58]. Therefore, no endothelial Gb3 deposition is expected in later-onset Fabry patients. The cardiac biopsies of our patients showed significant Gb3 accumulation in their cardiomyocytes but not in endothelial cells. It could indicate that IVS4 + 919G > A mutation is a true later-onset Fabry mutation.

Interestingly, a high frequency of hypertension (12 of 22; 54%) and hyperlipidemia (7 of 22; 32%) were noted among these cardiomyopathic patients with the IVS4 + 919G > A mutation. In this study, we did not check other genetic causes of hypertrophic cardiomyopathy. Actually, the exact causal relationship of these factors with hypertrophic cardiomyopathy in our patients has not established. Because most of our patients did not have significant renal impairment, the hypertension was not likely caused by Fabry renopathy. Several studies have indicated that in addition to renal impairment, hypertension could be caused by the imbalance of the autonomic nervous system in patients with Fabry disease [59]. Further studies are required to clarify the relationship between hypertension and the autonomic nervous imbalance in these patients. Furthermore, hypertension might be a predisposing factor to the development of hypertrophic cardiomyopathy for these later-onset Fabry disease patients, especially for the milder Fabry patients. The finding that four out of our five (80%) female patients had significant hypertension supports this finding. In addition, several IVS4 + 919G > A patients in our studies were found to be hypertensive. However, due to the small sample size in our study, we cannot make a definite conclusion regarding the relationship between hypertrophic cardiomyopathy and hypertension, more data will need to be collected and analyzed to clarify this point. Regarding predisposing factors for hypertrophic cardiomyopathy in later-onset Fabry patients, a recent important study, reported by Desnick *et al.*[60], revealed that as many as 20% of male and 25% of females with later onset Fabry mutations and hypertrophic cardiomyopathy concurrently have a pathogenic or likely pathogenic mutation of the HCM causing gene. Therefore, in addition to Gb3 accumulation, there should be some predisposing factors contributing to the pathogenesis of later-onset Fabry cardiomyopathy.

Regarding hyperlipidemia in Fabry patients, circulating Gb3 has been proved to be primary transported by LDL and HDL lipoproteins (approximately 60% and 30% respectively) [61]. Furthermore, hyperlipidemia was correlated with Fabry disease [62] and selective LDL-apheresis was shown to be an effective treatment in a Fabry patient with recurrent strokes [63]. However, the causal relationship between hyperlipidemia and hypertrophic cardiomyopathy of later-onset Fabry patients still needs to be clarified.

Interestingly, only scanty Gb3 accumulation could be found in the cardiomyocytes of our three patients who had received the longest period of ERT. This finding is quite different from several previous studies that showed the histological clearance of Gb3 accumulation from cardiomyocytes is usually poor [38,49,54,64]. Does this finding indicate that a longer duration of ERT still has efficacy for Gb3 clearance from cardiomyocytes of later-onset Fabry patients? However, without pre-ERT biopsies, it is impossible to claim ERT was able to clear Gb3 accumulation from the cardiomyocytes of these three patients. More cardiac biopsy data with pre-ERT and post-ERT should be collected to clarify this point.

Recent studies revealed that gadolinium-contrast cardiac MRI is a good method to assess myocardial fibrosis in Fabry disease [34,65-67]. In patients without fibrosis, ERT resulted in a significant reduction in left ventricular mass and improvement of myocardial function. In contrast, patients with mild or severe fibrosis showed a minor reduction in left ventricular hypertrophy and no improvement in myocardial function. In this study, one patient was found to have significant interstitial fibrosis among his cardiomyocytes by H&E staining of endomyocardial biopsy. However, no significant fibrosis was observed in his heart with late-enhancement technique by gadolinium-contrast cardiac MRI examination. On the other hand, 6 out of the 20 patients received gadolinium-contrast cardiac MRI examination in this study were found to have severe fibrosis (Table 1), but none of them were found to have any significant cardiac fibrosis in their endomyocardial biopsies. This finding indicates that the histological fibrosis of interventricular septum from right ventricular endomyocardial biopsy does not reflect the severity of the fibrosis of the whole heart; most of the fibrosis that significantly affect heart function is usually located in the left ventricular area in Fabry disease.

In this study, we analyzed the pathologic appearance of cardiomyocytes by endomyocardial biopsies in 22 atypical later-onset Fabry disease patients with the Chinese mutation (IVS4 + 919G > A). These data suggest that IVS4 + 919G > A is a real pathogenic mutation with typical Gb3 accumulation and hypertrophic changes in cardiomyocytes. No endothelial Gb3 accumulation was found in cardiac biopsies is consistent with the cardiac pathologic findings of later-onset Fabry disease.

## Conclusions

Fabry disease is a rare lysosomal disorder resulting from a genetic deficiency of α-Gal A. The present study used endomyocardial biopsies to confirm that the Chinese Fabry mutation (IVS4 + 919G > A) is a real pathogenic mutation.

## Competing interests

The authors declare that they have no competing interests.

## Authors’ contributions

TRH performed acquisition, statistical analysis, interpretation of data, and drafting of the manuscript. SHS, PCL, and WCY performed the endomyocardial biopsies. FPC and AHY participated in the histopathologic and electron microscopic interpretation. CFY, HCL, and HYL participated in data collection. CKH, HJG, YHH, HCL, and CCC carried out the biochemistric and genetic analysis. DMN and WCY participated in the design of the study, interpretation of the data and helped to draft the manuscript. All authors contributed in interpreting data, revising drafts of the manuscript and in the approval of the final manuscript.
